# Inclusion of Population-specific Reference Panel from India to the 1000 Genomes Phase 3 Panel Improves Imputation Accuracy

**DOI:** 10.1038/s41598-017-06905-6

**Published:** 2017-07-27

**Authors:** Meraj Ahmad, Anubhav Sinha, Sreya Ghosh, Vikrant Kumar, Sonia Davila, Chittaranjan S. Yajnik, Giriraj R. Chandak

**Affiliations:** 1 Genomic Research on Complex diseases (GRC Group), CSIR-Centre for Cellular and Molecular Biology, Hyderabad, Telangana 500 007 India; 2 Duke-NUS Medical School, 8 College Road, Singapore, 169857 Singapore; 3 SingHealth Duke-NUS Institute of Precision Medicine (PRISM), 20 College Road, The Academia, Discovery Tower, Level 7 Translational and Clinical Research Hub, Singapore, 169856 Singapore; 4 Diabetes Unit, King Edward Memorial Hospital and Research Centre, Rasta Peth, Pune, Maharashtra 411 011 India; 5 #5/1, 4th cross, Manjunatha Layout, Nagashettyhalli, 560094 Bengaluru India

## Abstract

Imputation is a computational method based on the principle of haplotype sharing allowing enrichment of genome-wide association study datasets. It depends on the haplotype structure of the population and density of the genotype data. The 1000 Genomes Project led to the generation of imputation reference panels which have been used globally. However, recent studies have shown that population-specific panels provide better enrichment of genome-wide variants. We compared the imputation accuracy using 1000 Genomes phase 3 reference panel and a panel generated from genome-wide data on 407 individuals from Western India (WIP). The concordance of imputed variants was cross-checked with next-generation re-sequencing data on a subset of genomic regions. Further, using the genome-wide data from 1880 individuals, we demonstrate that WIP works better than the 1000 Genomes phase 3 panel and when merged with it, significantly improves the imputation accuracy throughout the minor allele frequency range. We also show that imputation using only South Asian component of the 1000 Genomes phase 3 panel works as good as the merged panel, making it computationally less intensive job. Thus, our study stresses that imputation accuracy using 1000 Genomes phase 3 panel can be further improved by including population-specific reference panels from South Asia.

## Introduction

**Figure 1:**
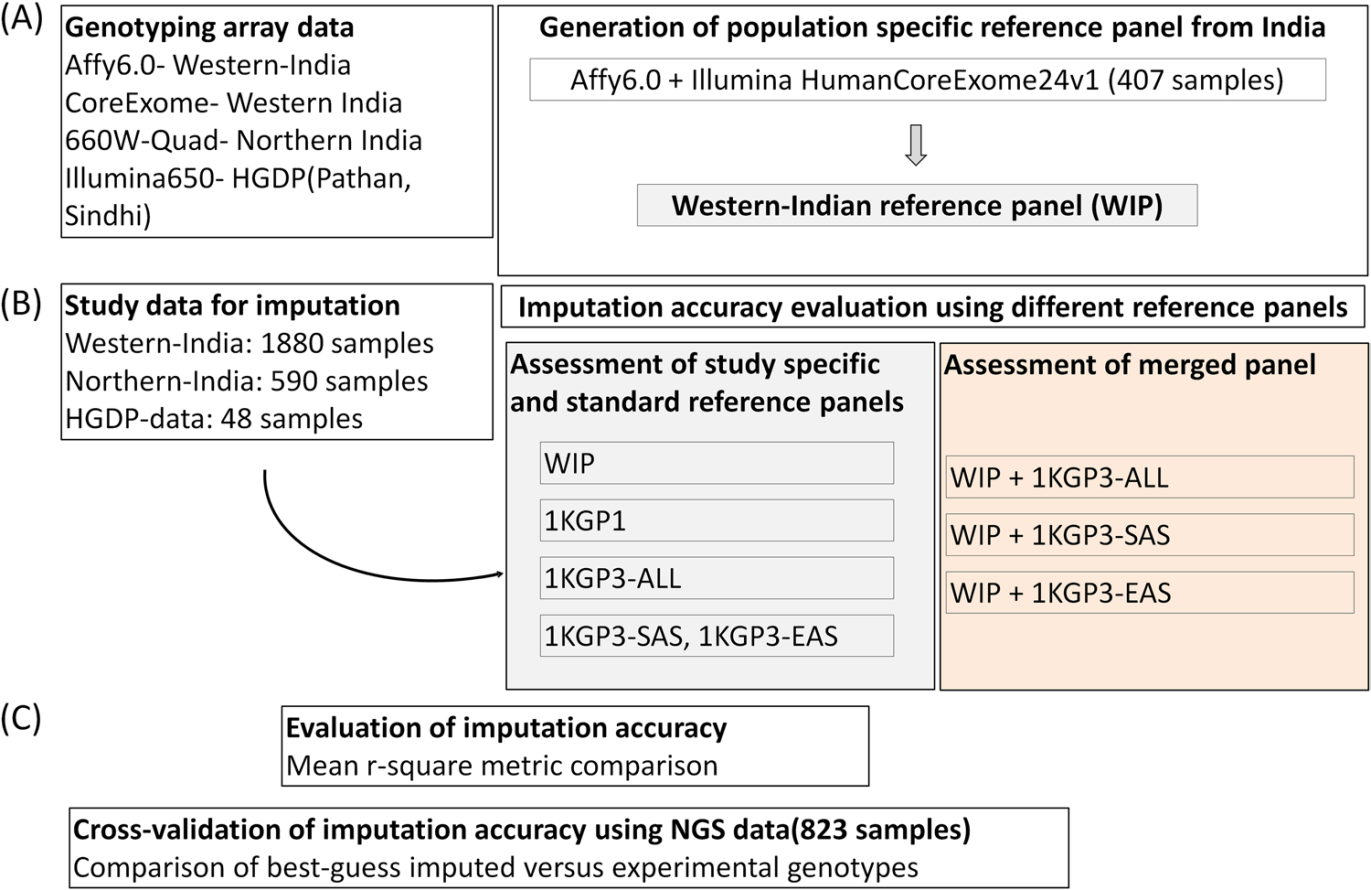
Schematic representation of the study design and analyses. Affy6.0 and Illumina HumanCoreExome data on 407 overlapping individuals from Western India was merged and used to generate the Western-Indian Reference Panel (WIP). SNPs from the Affy6.0 data on 1880 Western Indians, Human660W-Quad array data on 590 subjects from Northern India and HGDP data using Illumina 650K array on 48 Pathan and Sindhi subjects were imputed using different reference panels. The imputation accuracy was compared using r-square metric. Finally, cross-validation of imputation accuracy was performed on 823 samples having genotype data from HiSeq platform for the 3.57 Mb region and their imputed counterparts (imputed for Affy6.0 data). 1KGP1, The 1000 Genomes phase 1 panel; 1KGP3-ALL, The 1000 Genomes phase 3 panel with all 2504 samples; 1KGP3-SAS, The 1000 Genomes phase 3 panel with only South Asian component; 1KGP3-EAS, The 1000 Genomes phase 3 panel with only East Asian component; WIP+1KGP3-ALL, merged panel of WIP and 1KGP3-ALL; WIP+1KGP3-SAS, merged panel of WIP and 1KGP3-SAS; WIP+1KGP3-EAS, merged panel of WIP and 1KGP3-EAS.

Genome-wide Association Studies (GWASs) using high-density genotyping arrays have facilitated the understanding of genetic basis of many complex diseases and related intermediate traits^1^. This has led to the identification of hundreds of risk loci for certain diseases like type 2 diabetes (T2D); a majority of these loci have also been replicated in populations other than the one where they were identified in the first place^2^. Many genomic regions have also been fine-mapped to uncover the potential causal variant from among a pool of variants which are in linkage disequilibrium (LD) with one another in a haplotype block. The single nucleotide polymorphism (SNP) density of the genotyping arrays varies and so does the availability of genotype data across various association studies. Imputation is a useful and cost-effective computational strategy based on the pattern of LD structure and sharing of haplotype stretches among individuals. It allows analysing a larger number of variants without genotyping them directly. Thus, it helps to perform deeper genetic investigation using limited resources^3^ and increases the power of GWAS, meta-analyses and fine mapping studies. Researchers across the world have been using The 1000 Genomes phase 1 reference panel (1KGP1) for imputation with reasonable accuracy, however, the absence of genomic information on various populations raises concerns about its global suitability^4^. Recently, several novel loci have been reported to be associated with disease phenotypes in specific populations^5–7^, which brings GWASs to a phase where population-specific loci expand the understanding of the mechanisms and pathways linked to a disease. It is thus important to have appropriate reference panel for accurate imputation since features such as haplotype structure, presence of population-specific variants and altered frequency of variants influence the imputation quality and genomic coverage of the imputed variants^3^. Two independent studies using Japanese population reference panel (1KJPN) from 1070 Japanese individuals and Genome of Netherlands (GoNL) panel from 769 Dutch individuals have been shown to add to the imputation accuracy while using the 1KGP1 panel^8, 9^. Recently, a reference panel of 64,976 haplotypes of predominantly European ancestry has been made available which enables accurate genotype imputation of variants with minor allele frequency (MAF) as low as 0.1% for European datasets^10^. India has a population size of more than 1.2 billion and comprises several thousand endogamous sub-populations^11–14^. Using high-throughput data, genetic evidence has provided evidence of the different origins of various populations and demonstrated the existence of various founder populations^13^. Thus, the Indian population structure makes it well-suited to explore population-specific genetic risk loci but such efforts have hardly been undertaken. One of the major concerns is false positive disease associations due to population stratification among individuals recruited from the same region since genetically different populations may live in close geographical proximity due to social and cultural traditions while maintaining their genetic isolation. The second and equally important factor is the absence of adequate representation of Indian population in the reference panels used for imputation analysis. The 1KGP1 has no representation while The 1000 Genomes phase 3 reference panel (referred as 1KGP3-ALL to denote the full panel hereafter) includes 5 populations related to the Indian subcontinent (though none from India) and hence is expected to provide better imputation accuracy than 1KGP1^4, 15^. We evaluated the imputation accuracy of GWAS data generated on individuals of Indo-European ethnicity from Western India, using 1KGP1 and 1KGP3-ALL panels. We further generated an Indian reference panel combining the Affy 6.0 data and Illumina CoreExomeBeadchip data and queried whether this panel in isolation or in combination with 1KGP3-ALL offers greater imputation accuracy in 3 different GWAS datasets (Fig. 1). Finally, we validated the imputation accuracy using targeted next-generation sequencing (NGS) data on a subset of genomic regions. Our findings provide evidence that in genetic association studies, a larger contribution from Indian populations will help in better understanding of genetic diseases. Large-scale genomic and sequencing studies are needed to exploit the potential offered by the unique Indian population structure.

## Results and Discussion

The 1000 Genomes Project was launched to provide deeper characterization of the human genome sequence variations^15^. The 1KGP1 included 1092 individuals from 14 populations and the 1KGP3-ALL has data from 2504 individuals from 27 world populations^4^. We investigated the utility of a newly generated Western-Indian reference panel (WIP) by comparing the imputation accuracy with 1KGP3-ALL and observed that the WIP confers a significant improvement in imputation accuracy across the minor allele frequency when merged with the 1KGP3-ALL panel (p < 0.05). We further demonstrate that using only the South Asian component of 1000 Genomes phase 3 dataset provides imputed data of similar quality as 1KGP3-ALL and may be used for imputation analysis using lesser computational resources. Based on our results, we make several recommendations for imputation/association analysis of GWAS data in South Asians, especially in Indians.

### The 1000 Genomes phase 3 panel yields better imputation accuracy of GWAS data than the 1000 Genomes phase 1 panel

The SNP data on chromosome 20 in Affy 6.0 (18416 SNPs) on 1880 subjects, were utilized to perform imputation by masked/imputed genotype approach using IMPUTE2^16^. First, we compared 1KGP1 with 1KGP3-ALL at khap = 1000 and observed better imputation accuracy using the 1KGP3-ALL panel as measured by mean r-square metric (Supplementary Figure 1). As expected, the 1KGP3-ALL imputed more SNPs than the 1KGP1 panel (18267 vs. 17103 SNPs). Next, we queried for optimum khap parameter for the 1KGP3-ALL panel. It was interesting to note that khap 500, the default value suggested by the IMPUTE2 team and khap 1000, used by the 1000 Genomes project for imputation assessment performed sub-optimally for our samples^4^. The accuracy of imputation improved with increasing khap value till 3000 which is consistent with an earlier study that suggested the need for an optimal number of “surrogate family” haplotypes for best imputation^17, 18^ (Supplementary Figure 2). The khap parameter determines the number of reference haplotypes to be used in the “custom” reference panel for each study individual and a larger khap may be needed for populations with higher genetic diversity or admixed populations like Indians. Further comparisons in the study were performed at khap 3000. The added accuracy while using 1KGP3-ALL can be assigned to the inclusion of 5 populations of South Asian Ancestry (SAS; BEB-Bengali in Bangladesh; GIH-Gujarati Indian in Houston, TX; ITU-Indian Telugu in the UK; PJL-Punjabi in Lahore, Pakistan; STU-Sri Lankan Tamil in the UK) in the 1KGP3-ALL panel. Thus, addition of ethnicity-specific genomic data in the reference panel improves the imputation accuracy.

### Imputation Evaluation of Western-Indian Reference Panel Versus the 1000 Genomes Reference Panels

**Figure 2:**
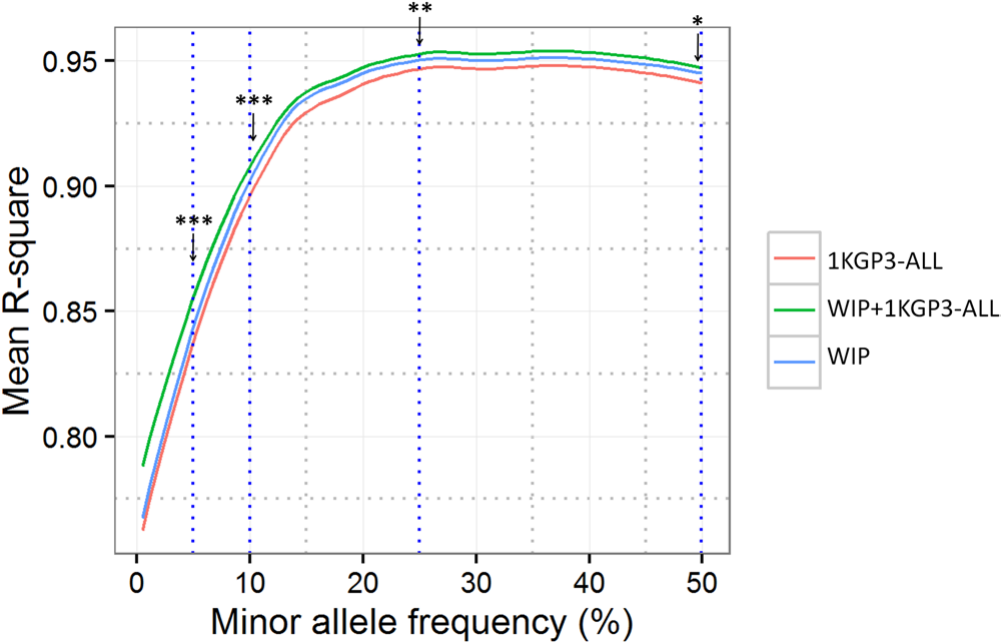
Evaluation of population-specific reference panel for imputation accuracy. Affy6.0 SNPs from 1880 individuals from Western India were imputed at khap 3000 using 3 different reference panels: The 1000 Genomes Phase 3 (1KGP3-ALL), Western-Indian reference panel (WIP) and mergedWestern-Indian-1KGP3-ALL (WIP+1KGP3-ALL). Average r-square values were plotted against each minor allele frequency (MAF) bin. Two-tailed paired-end TTEST was performed for the mean r-square values at given MAF-bins between 1KGP3-ALL and WIP+1KGP3-ALL panel imputed SNPs. ‘p’ values of <0.001, <0.01 and <0.05 are indicated by ***, ** and * respectively. Results are restricted to SNPs on chromosome 20 only.

We generated the Western-Indian Reference Panel, a population-specific reference panel from India by combining the Affy6.0 chip data with Illumina HumanCoreExome data on 407 individuals from the Pune Maternal Nutrition Study (PMNS)^19^. The combined SNP dataset includes 931,371 high quality autosomal SNPs. The WIP was further merged with 1KGP3-ALL using IMPUTE2 to generate the WIP+1KGP3-ALL panel^20, 21^ (see Methods). SNPs on chromosome 20 in Affy6.0 data on remaining 1880 individuals were imputed using the above reference panels. Comparison of the r-square values obtained from IMPUTE2 in leave-one-out scenario averaged for each MAF bin for SNPs common between the imputed datasets (18266 SNPs) shows that the WIP confers marginal enhancement in imputation performance than the 1KGP3-ALL panel (Fig. 2). The accuracy is further enhanced significantly across the minor allele frequency spectrum when the combined panel WIP+1KGP3-ALL is used for imputation analysis (p < 0.05 for >93% MAF bins). Our results are consistent with the earlier reports where the use of population-specific panels like 1KJPN from Japan and GoNL from Netherlands improved the imputation accuracy across the MAF spectrum^8, 20, 22^. However, the difference in mean r-square values did not show as big difference for low-MAF variants as was observed in the 1KJPN^8^ (Fig. 2). Similar analysis using imputation quality score (IQS) did not validate these observations suggesting that they could be chance agreements. This may be related to a small number of low-frequency variants in our limited dataset compared to the 1KJPN which used the WGS data for making reference panel. IQS is not commonly used for imputation accuracy measurements and comparison of IQS with other widely used methods like squared correlation and Beagle-r-square shows large discrepancies over all MAF values^23^. We speculate that these discrepancies occur because of the assumptions made for Cohen’s kappa statistics used in IQS^24^. However, despite limited SNP density, this population-specific reference panel offers better imputation accuracy likely due to the inclusion of Indian-specific haplotype when the WIP is merged to 1KGP3-ALL. This suggests that there is still scope to improve the 1000 Genomes phase 3 panel and provide better imputation performance for other ethnic populations of the world. A truly comprehensive reference panel using whole-genome sequence data from Indians would be ideal to fill these lacunae in the disease genomics studies in South Asia.

### Assessing Concordance of Imputed Genotypes using Next Generation Sequencing Data and Performance of The1000 Genomes South Asian Ancestry Samples

**Figure 3:**
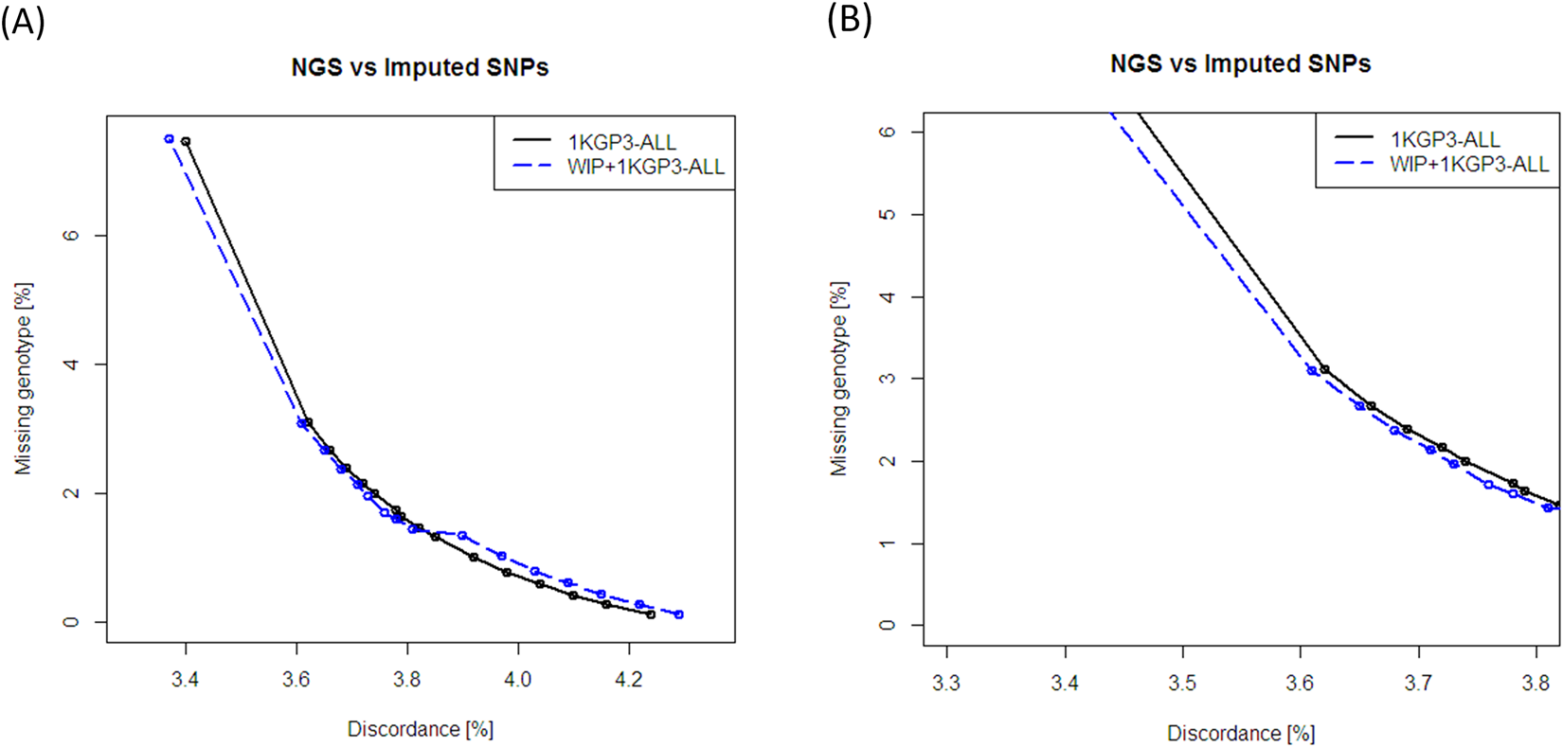
Validation of imputation performance using genotypes from targeted next-generation sequencing. The imputed genotypes in Affy6.0 data on 823 individuals generated using different panels were compared with the genotypes at 18979 common SNPs from targeted NGS of 3.57 Mb region. The imputation performance is illustrated by the percentage discordance (X-axis) plotted against percentage missing genotypes (Y-axis) for the SNPs common to the imputed and NGS genotype datasets. The figure shows the (**A**) full range of results corresponding to the probability thresholds ranging from 0.33 to 1.00 and (**B**) magnified results for probability thresholds near 0.90 and above for better comparison. 1KGP3-ALL, The 1000 Genomes phase 3 reference panel; WIP, Western-Indian reference panel; WIP+1KGP3-ALL, merged panel of WIP and 1KGP3-ALL; NGS, next generation sequencing; SNPs, single nucleotide polymorphisms.

Imputation performance was assessed using a moderately dense GWAS array SNPs by masked/imputed genotype method but a comparison of the imputed SNPs in certain genomic regions with much denser experimental genotype data available from NGS is desirable. Hence, we cross-checked the imputed data by comparing concordance between imputed genotypes generated using above panels and the direct genotypes obtained through targeted NGS for a 3.57 Mb region spanning chromosomes 3, 5 and 10 on 823 subjects among the 1880 individuals. There were 18979 common SNPs between the three datasets which were compared using vcftools^25^. The imputed calls are assigned hard call genotypes based on probability values obtained for the three possible genotypes (AA, AB and BB) and those not passing the probability threshold are ascribed as missing genotypes. Both the error rate in imputation calling between imputed genotype calls and observed NGS calls (percentage discordance), and the percentage of missing genotypes (missingness) were considered, which gives an idea about the imputation error rate (Fig. 3). For a given missingness threshold, percentage discordance was lesser for WIP+1KGP3-ALL as compared to the 1KGP3-ALL panel indicating that concordance between imputed and true genotypes is higher with the WIP+1KGP3-ALL panel (Fig. 3). For example, at a genotype probability threshold of 0.92, the missingness is 1.71% in WIP+1KGP3-ALL as compared to 1.74% in 1KGP3-ALL while under the same conditions the discordance is 3.76% and 3.78% respectively (Fig. 3). This is important since SNPs above a certain missingness threshold are filtered out from the high throughput data and only those which pass are taken forward for further analysis. It is worth noting that out of 18979 imputed SNPs common between the merged panel and 1KGP3-ALL panel, a higher number of SNPs pass a given info score threshold (n = 12258 and 11992 SNPS at info cut off >0.7, for the merged and 1KGP3-ALL panels respectively) (Supplementary Table 1).

**Figure 4:**
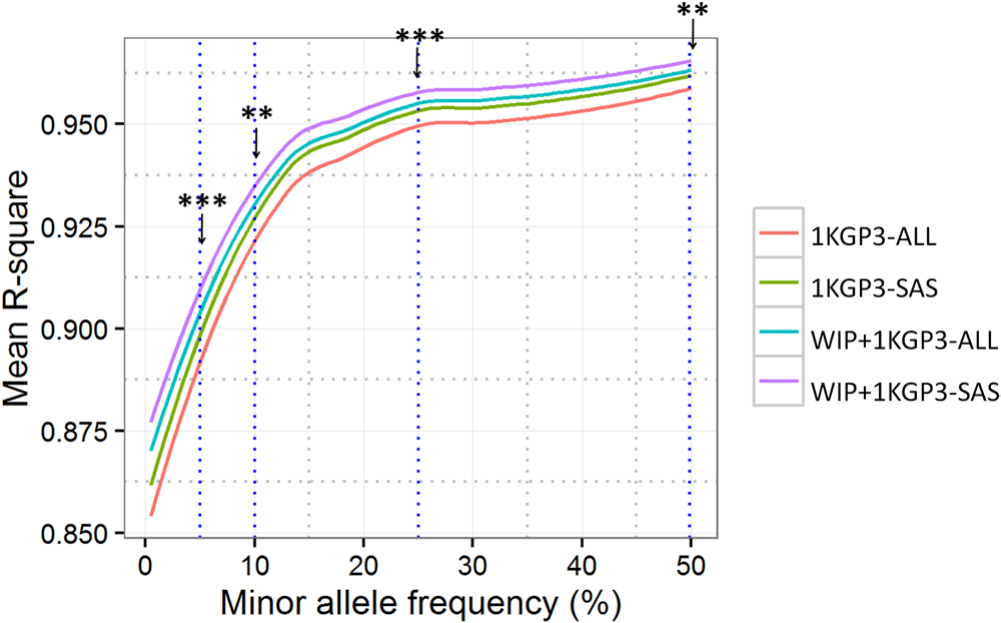
Comparison of imputation accuracy using 1000 Genomes phase 3 (1KGP3-ALL), Western-Indian panel (WIP) and 1000 Genomes phase3-SASonly (1KGP3-SAS) reference panels. Affy 6.0 SNPs from 1880 individuals from Western India were imputed at khap 3000 using 1KGP3-ALL and 1KGP3-SAS and average r-square values were plotted against each minor allele frequency (MAF) bin. Two-tailed paired-end TTEST was conducted for the mean r-square values at given MAF-bins between 1KGP3-ALL and WIP+1KGP3-SAS panel imputed SNPs. ‘p’ values of <0.001 and 0.01 are indicated by *** and ** respectively. Results are restricted to chromosome 20 only. 1KGP3-ALL, The 1000 Genomes phase 3 reference panel; 1KGP3-SAS, The 1000 Genomes phase 3 panel with only South Asian component; WIP+1KGP3-ALL, merged panel of WIP and 1KGP3-ALL; WIP+1KGP3-SAS, merged panel of WIP and 1KGP3-SAS.

In view of the observation that inclusion of ethnicity- and population-specific reference panels improve the imputation accuracy, we investigated if using only the data from the South Asian populations would have similar results as the 1KGP3-ALL panel. We generated two more reference panels, 1KGP3-SAS and 1KGP3-EAS comprising data from 489 and 504 individuals from South Asian and East Asian ancestry respectively from 1KGP3-ALL (see Methods). These panels were further combined with the WIP to get merged panels (WIP+1KGP3-SAS and WIP+1KGP3-EAS) respectively. Indeed, the imputation accuracy for 1KGP3-SAS reference panel was marginally better than the 1KGP3-ALL panel (p < 0.05 for 48.7% MAF bins) (Fig. 4); but was significantly lower for the 1KGP3-EAS panel (Supplementary Figure 3). It was equally interesting to note that the imputation performance using the merged WIP+1KGP3-SAS panel was equally good as WIP+1KGP3-ALL panel and the exercise was completed in a shorter time frame. This suggests that using genetically close populations in a population-specific reference panel provides better imputation performance than using a cosmopolitan reference panel. In order to independently confirm the above observation, we imputed common variants (18272 SNPs on chromosome 20) from only the ITU component of 1000 Genomes phase 3 data using three different panels where ITU dataset was removed; 1KGP3-ALL, 1KGP3-SAS, WIP+1KGP3-SAS. We observed that the “WIP+1KGP3-SAS-without ITU” panel works better than the other two panels (p < 0.05 for 41.7% MAF bins) (Suplementary Figure 4). Thus, a marginally better performance by a smaller subset of the 1KGP3-ALL panel against the full panel is a true phenomenon, although the significance may vary depending on the population used for imputation. Thus, we demonstrate that there may not be a need for a big cosmopolitan panel and a reference panel made from a set of ethnicity-specific populations may be sufficient for imputation analysis.

### Evaluation of Imputation Accuracy in Other South Asian Populations

**Figure 5:**
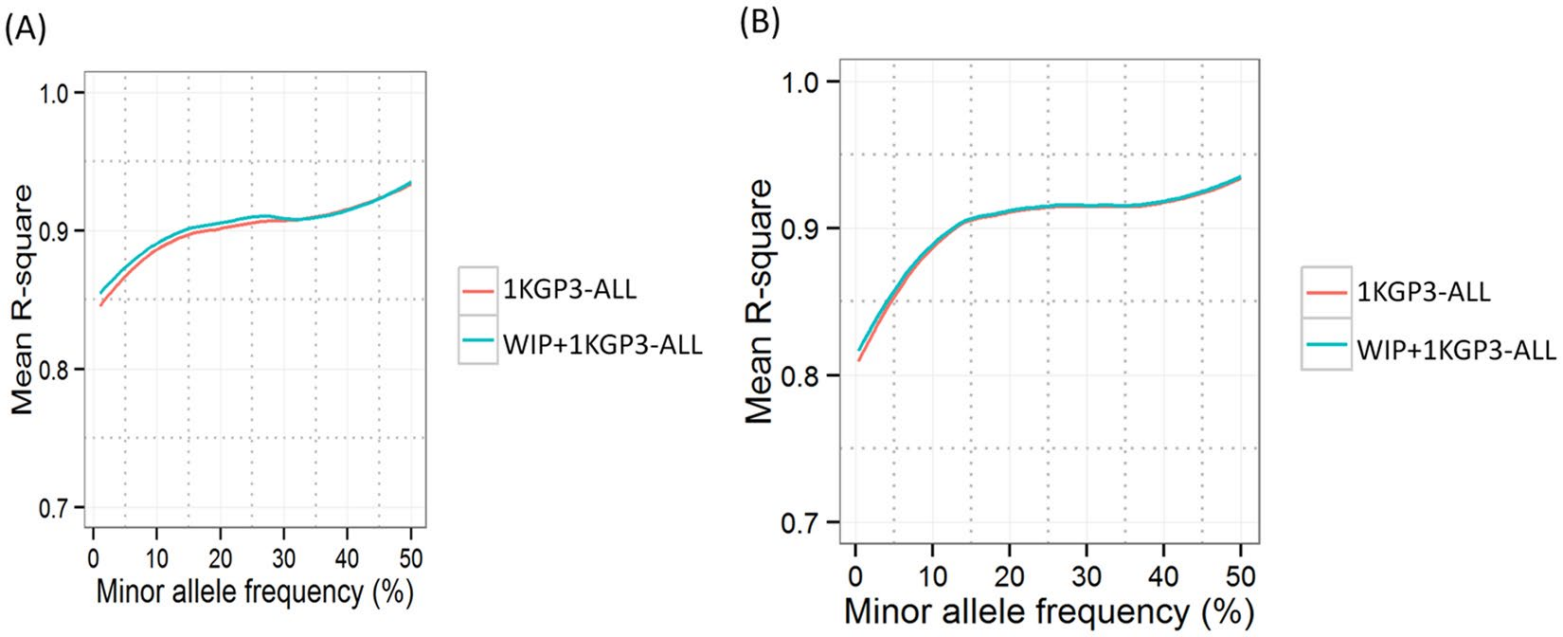
Comparison of imputation performance of data from other populations generated using 1KGP3-ALL and WIP+1KGP3-ALL reference panels. The imputation performance of the reference panels, 1KGP3-ALL and WIP+1KGP3-ALL was evaluated by comparing the imputed SNPs from other South Asian populations (Pathan and Sindhi from Human Genome Diversity Project (HGDP), and North-Indian individuals). (**A**) SNPs from HGDP data on Pathan and Sindhi populations (n = 48, 12494 SNPs) and (**B**) SNPs on North-Indian samples (13746 SNPs, n = 590). Results are restricted to chromosome 20 only. 1KGP3-ALL, The 1000 Genomes phase 3 reference panel; WIP+1KGP3-ALL, merged panel of WIP and 1KGP3-ALL.

Finally, we investigated the utility of the WIP and other reference panels in few other populations from South Asian region. SNPs on chromosome 20 from Illumina Human650K array data (12494 SNPs) on 48 Pathan and Sindhi subjects (as available from the HGDP-CEPH project)^26^ were imputed. As expected, imputation using the WIP panel showed poor results because this panel comes from a specific Western India population having a different genetic structure from Pathan and Sindhi populations and has less genomic coverage of SNPs. However, we observed a marginal and statistically significant (p < 0.05 for 4.5% MAF bins) improvement in the mean r-square values over few MAF bins for the WIP+1KGP3-ALL as compared to the 1KGP3-ALL panel (Fig. 5A). Similar to the HGDP-CEPH data, comparison with another cohort from Northern India^27^ also showed marginal and statistically significant (p < 0.05 for 6.8% MAF bins) improvement in the average r-square value (Fig. 5B) in several MAF bins (especially in the lower MAF range) for WIP+1KGP3-ALL than the 1KGP3-ALL panel. It may be worth mentioning that the WIP+1KGP3-SAS panel performed similar to the WIP+1KGP3-ALL panel (Supplementary Figure 5). It is well established that haplotype stretch matching is maximised by using population specific reference panels and inadequate representation of genetically diverse populations in a cosmopolitan panel warrants inclusion of population specific panel to achieve greater accuracy^28, 29^. Hancock *et. al*. noted that imputation quality is reduced by addition of more distantly related reference populations which is consistent with our findings^28^. Moreover, though none of the imputation methods explicitly account for admixture, the underlying models differ substantially in their ability to capture patterns of haplotype diversity created by admixture and diversity in genetic architecture which further substantiates the need for population specific reference panels^30^. Haplotype diversity associated with South Asian ancestry components is known to be significantly higher than that of the components dominating West Eurasian ancestry palette and region-specific signals of positive selection and genetic structuring among Indian populations has been well documented^31^. This study further stresses on the existence of population substructure among the Indian populations and on the need for a more comprehensive reference panel from Indian populations with denser genotype information based on whole genome sequence data. Such a panel could then be applied to other populations and will further enhance the imputation accuracy.

In summary, we show that use of population-specific reference panel leads to better imputation which can improve results from association studies. We also find that despite the inclusion of 5 populations related to the Indian subcontinent in the 1000 Genomes phase 3 reference panel, there is still scope for the improvement of the imputation accuracy as demonstrated using a reference panel made using limited genotype data from a cohort from Western India. Owing to second highest genetic diversity in India after African populations, including more Indian samples from different ethnic backgrounds and sub-populations may help to enhance the accuracy of imputation. We also demonstrate that a cosmopolitan reference panel (1KGP3-ALL) is dispensable if we combine South Asian populations to make a reference panel (WIP+1KGP3-SAS) for imputation of samples from South Asia and more populations from South India or East India may further enrich the reference panel. This will further help to improve and broaden the scope of genetic association studies in South Asian populations and especially Indian diaspora which has been proposed to have an excess of genetic diseases^13, 32^ and unique population structure due to socio-cultural and historical peculiarities.

## Methods

### Samples and Datasets

Genome-wide SNP data generated using Affy 6.0 array chip (Affymetrix, CA, USA) on 2410 individuals comprising parents of the children in the Pune Maternal Nutrition Study^19^ and patients in the Wellgen study^33^; Illumina HumanCoreExome data on 407 individuals from the PMNS, Illumina Human660W-Quad BeadChip data on 590 individuals from North India^27^ and the Human Genome Diversity Panel (HGDP) data^26, 34^ on Pathan and Sindhi populations (n = 48) were utilised for this study. Targeted NGS data on 3.57 Mb region was used for cross-validation of the imputation performance. All the subjects gave written informed consent and the study was approved by the Institutional Ethics Committee of Centre for Cellular and Molecular Biology, Hyderabad and all experiments were performed following guidelines for human research by Indian Council of Medical Research, Government of India, New Delhi.

### Genotyping and Quality Control Analysis for Affy 6.0 and Illumina HumanCoreExome-24v1.0 Array Datasets

Data from Affy 6.0 chips was collated and SNPs with call rate <97%, MAF <0.5%, or those with Hardy-Weinberg equilibrium (HWE) p < 1 × 10^−6^ were removed. Reported gender was verified using two methods, X chromosome heterozygosity and average log ratio of chromosome X and Y intensity and 27 mismatch samples were excluded. Cryptic relatedness and duplicate samples were identified using identity by descent methods implemented in PLINK v1.07 (http://pngu.mgh.harvard.edu/purcell/plink/)^35^, and 104 individuals were excluded using PI_HAT ≥0.45. Overall, 123 samples were excluded for multiple overlapping reasons leaving 2287 individuals with 709687 autosomal SNPs for further analysis.

Intensity files from Illumina HumanCoreExome-24v1.0 on 638 subjects from the PMNS were analysed using Genome Studio-v2011.1 (Genotyping module-v1.9.4). The samples with a call rate <98% were removed. In brief, markers with p10GC <0.38, low intensity (AB R mean ≤0.25), call frequency <0.95, HWE P < 1 × 10^−6^ were removed. Markers with bad clustering, heterozygote excess, poor separation of theta axis and with bad cluster separation were manually curated and bad calls were filtered out as recommended by Illumina. The data on 407 subjects was used for further quality control filtering and the SNP data was converted into the plus strand, hg19/build37 format. Monomorphic and non-autosomal SNPs were also excluded, leaving 303925 available SNPs on 407 samples for further analysis.

### Targeted Re-sequencing

Based on an earlier GWAS, a 3.57 Mb target genomic region encompassing 37 genes on chromosomes 3, 5 and 10 was sequenced for 101 bp paired-end reads on 1055 samples from PMNS and Wellgen using HiSeq2000. The majority of samples (95%) had >30X depth of coverage and >90% bases were covered at least 4X. We mapped individual sequence reads to the human reference genome (NCBI Build 37, hg19) with Burrows-Wheeler Aligner. After recalibration and realignment, the consensus genotypes were called using UnifiedGenotyper in GATKv2.3–9 and annotation of the variants was performed using ANNOVAR (May 2012 release). A total of 45068 SNPs passed the QC analysis and the data for 823 samples common to Affy6.0 individuals were used for further analysis.

### Reference panels used in the study

Publicly available reference panel datasets for The 1000 Genomes phase 1 (1KGP1; https://mathgen.stats.ox.ac.uk/impute/data_download_1000G_phase1_integrated_SHAPEIT2_16-06-14.html) and The 1000 Genomes phase 3 (1KGP3-ALL; https://mathgen.stats.ox.ac.uk/impute/1000GP_Phase3.html) were downloaded. From the 1KGP3-ALL panel, South Asian and East Asian population-specific reference panels (1KGP3-SAS and 1KGP3-EAS respectively) were generated by subsetting the phased haplotypes from the 1KGP3-ALL panel using IMPUTE2 (commands provided in Supplementary Note 1). SNPs from HumanCoreExome-24v1.0 and Affy 6.0 were pooled (931,371 autosomal high quality SNPs) to generate the Western Indian Reference Panel (WIP), which was merged with 1KGP3-ALL to create the combined WIP+1KGP3-ALL panel (commands given in Supplementary Note 2). The two reference panels were merged using “-merge_ref_panels_output_ref” option in IMPUTE2, which imputes the variants that are specific to one reference panel into the other panel and thereafter creates a new reference panel with the union of the variants from the two panels. As the two reference panels have been imputed up to the union of their variants, the imputed haplotypes are treated as known haplotypes (*i.e*., take the best-guess haplotypes). The common SNPs from 1000 Genomes ITU population were extracted from the open access data and imputed using panels where ITU samples were removed from the reference panels.

### Evaluation of Imputation Accuracy using above Reference Panels

The study sample datasets were split chromosome-wise, pre-phased by SHAPEITv2 and imputed by IMPUTEv2.3.2^16, 17, 21^ using different reference panels as discussed above. IMPUTE2 masks the genotypes of one variant at a time in the study data and then imputes the masked genotypes using information from the reference data and the nearby study variants. For IMPUTE2 options, Ne and buffer values were set to 20000 and 1000 kb, respectively. Each chromosome was split into chunks of 5 Mb region and we utilized chromosome 20 for assessment of imputation accuracy using khap = 3000. Imputation accuracy can be measured using different statistics such as r-squared correlation, concordance rate and imputation quality score (IQS). All of them produce similar assessments for common variants but IQS is especially recommended for rare and low frequency variants since it appropriately adjusts for chance agreements^23^. Hence, we used r-square for all comparisons and cross-checked the results of rare and low frequency variants using IQS. The r-square is the information metric calculated as the squared correlation between input and masked/imputed genotypes at a SNP and is shown as the “r2_type0” column in the _info file. It takes values between 0 and 1, where values near 1 indicate that a SNP has been imputed with high certainty^3^. Using R- packages, the r-square metric from the _info file averaged for each MAF bin (bin-size 0.1% MAF) was plotted for each imputed dataset^3, 16^. The statistical significance of the differences in mean r-square values was analysed using two-tailed paired-end t-test taking all the r-square values at a given MAF bin for SNPs imputed using two different reference panels. IQS is based on Cohen’s kappa statistic and assesses the agreement between two methods while adjusting for chance agreement particularly for uncommon SNPs (MAF <5%)^36^. Concordance is ascertained based on best-guess genotype rather than imputed probabilities and we used concordance rate to further validate the imputed genotypes by comparing them with NGS genotypes on a subset of regions from chromosomes 3, 5 and 10 over a range of info thresholds (0.3 to 1.0). The discordant genotypes were compared using “–diff-site-discordance” utility in vcftools^16^ and the percentage discordance and missingness calculated from absolute number of SNPs at probability thresholds (0.33 to 1.00) were plotted.

## Electronic supplementary material

Supplementary Information
