# Supplementary material for: Inclusion of Population-specific Reference Panel from India to the 1000 Genomes Phase 3 Panel Improves Imputation Accuracy
Source: Sci Rep. 2017 Jul 27;7:6733. doi: 10.1038/s41598-017-06905-6 (PMC5532257; doi:10.1038/s41598-017-06905-6)
Supplement: Supplementary file 1 — Supplementary Information [file 41598_2017_6905_MOESM1_ESM.doc]

**Inclusion of Population-specific Reference Panel from India to the**

**1000 Genomes Phase 3 Panel Improves Imputation Accuracy**

Meraj Ahmad^1^, Anubhav Sinha^1*^, Sreya Ghosh^1^, Vikrant Kumar^2^,

Sonia Davila^2,3^, Chittaranjan S Yajnik^4^, Giriraj R Chandak^1#^

# *G*enome *R*esearch on *C*omplex diseases (GRC Group), CSIR-Centre for Cellular and Molecular Biology, Hyderabad, Telangana, 500 007, India.

1. Duke-NUS Medical School, 8 College Road, 169857, Singapore.

# SingHealth Duke-NUS Institute of Precision Medicine (PRISM), 20 College Road, The Academia, Discovery Tower, Level 7 Translational and Clinical Research Hub, Singapore 169856

# Diabetes Unit, King Edward Memorial Hospital and Research Centre, Rasta Peth, Pune, Maharashtra, 411 011, India.

# *, Current address - #5/1, 4th cross, Manjunatha Layout, Nagashettyhalli, 560094, Bengaluru, India.

**Address for correspondence**

#Giriraj R Chandak

Scientist and Group Leader

CSIR-Centre for Cellular and Molecular Biology, Hyderabad, Telangana, 500 007. India

Tel: +914027192748; Fax: +914027160591

E-Mail: chandakgrc@ccmb.res.in

**Supplementary Figure 1: Comparison of imputation accuracy of the 1000 Genomes phase 1 and phase 3 reference panels.** Imputation was carried out for Affy 6.0 array SNP data on 1880 individuals from Western India using 1KGP1 and 1KGP3-ALL panels at khap=1000 and the average r-square values for each minor allele frequency (MAF) bin were plotted. The MAF bin size is 0.1%. Accuracy assessment for imputed genotypes was done using the r-square metric which is squared correlation between real and masked/imputed genotypes. Two-tailed paired-end ‘T’ TEST was performed for mean r-square values at given MAF-bins between 1KGP1 and 1KGP3-ALL panel imputed SNPs. ‘p’ values of <0.001, <0.01 and <0.05 are indicated by ***, ** and * respectively. Results are restricted to SNPs on chromosome 20 only. 1KGP1, The 1000 Genomes phase 1 panel; 1KGP3-ALL, The 1000 Genomes phase 3 panel with all 2504 samples.

**Supplementary Figure 2: Imputation performance using the 1000 Genomes phase 3 reference panel (1KGP3-ALL) at different khap parameters.** Imputation was carried out for Affy 6.0 array SNP data on 1880 individuals from Western India. Mean r-square values for masked imputation analysis were plotted against the minor allele frequency bins for khap of 500, 1000, 2000, 3000 and 5000. The results are restricted to chromosome 20.

**Supplementary Figure 3: Comparison of imputation performance of the 1000 Genomes phase 3, WIP and 1KGP3-EAS only reference panels.** Imputation was carried out for Affy 6.0 array SNP data on 1880 individuals from Western India using different reference panels and mean r-square values were plotted against the minor allele frequency bins. The results are restricted to chromosome 20. 1KGP3-ALL, the 1000 Genomes phase 3 panel with all 2504 samples; WIP, Western Indian reference panel; WIP+1KGP3-ALL, merged panel of WIP and 1KGP3-ALL; 1KGP3-EAS, the 1000 Genomes phase 3 panel with only East Asian component; WIP+1KGP3-EAS, merged panel of WIP and 1KGP3-EAS.

**
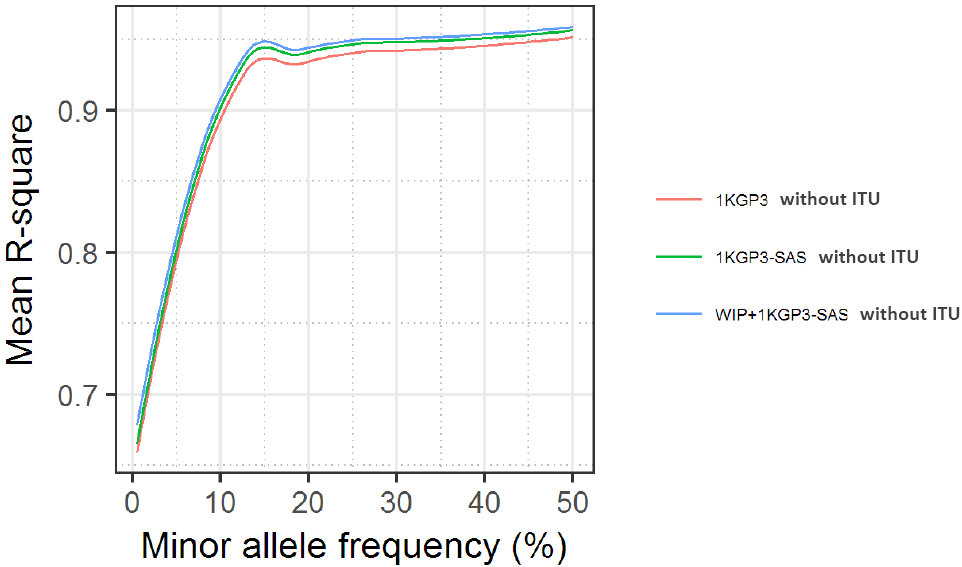
**

**Supplementary Figure 4: Comparison of the imputation performance of the 1000 Genomes phase 3 reference panel with its smaller subset panels along with WIP panel.** Imputation was carried out for the 1000 Genomes ITU dataset and SNPs common to our Affy 6.0 dataset on chromosome 20 were imputed usingthree reference panels (1KGP3-ALL, 1KGP3-SAS, WIP+1KGP3-SAS respectively) without ITU population in the panels. Mean r-square values for masked imputation analysis were plotted against the minor allele frequency bins. 1KGP3 without ITU, the 1000 Genomes phase 3 panel without ITU data; 1KGP3-SAS without ITU, The 1000 Genomes phase 3 panel with only South Asian component and without ITU data; WIP+1KGP3-SAS without ITU, merged panel of WIP and 1KGP3-SAS without ITU data.

**Supplementary Figure 5: Comparison of imputation performance using the 1000 Genomes phase 3 reference panel (1KGP3-ALL) with 1KGphase3-SAS only panel (1KGP3-SAS).** Imputation was carried out for (A) Human660W Quad-Beadchip SNPs on Pathan and Sindhi (13746 SNPs), and (B) Human 660w-Quad array SNPs on North-Indian samples (12326 SNPs) using WIP+1KGP3-ALL and WIP+1KGP3-SAS reference panels. Mean r-square values for masked imputation analysis were plotted against the minor allele frequency bins. The results are restricted to chromosome 20. WIP+1KGP3-ALL, merged panel of WIP and 1KGP3-ALL; 1KGP3-SAS, the 1000 Genomes phase 3 panel with only South Asian component; WIP+1KGP3-SAS, merged panel of WIP and 1KGP3-SAS.

**Supplementary Table 1.** **Comparison of imputation quality using info metric between 1KGP3-ALL and WIP+1KGP3-ALL panels.**

| **Info score cut off** | **1KGP3-ALL panel** | **WIP+1KGP3-ALL panel** |
| --- | --- | --- |
| > 0.3 | 16064 | 16148 |
| > 0.4 | 15412 | 15598 |
| > 0.5 | 14542 | 14749 |
| > 0.6 | 13426 | 13733 |
| > 0.7 | 11992 | 12258 |
| > 0.8 | 10359 | 10489 |
| > 0.9 | 8536 | 8587 |

The imputed genotypes in Affy6.0 data on 823 individuals generated using the above two panels were compared with the genotypes at 18979 common SNPs in the 3.57 Mb NGS region and the number of SNPs passing different info score thresholds are represented. 1KGP3-ALL, The 1000 Genomes phase 3 reference panel; WIP+1KGP3-ALL, merged panel of WIP and 1KGP3-ALL; NGS, next generation sequencing; SNPs, single nucleotide polymorphisms.

**Supplementary Note 1:**

**Commands for making subsets from 1000 Genomes phase 3 reference panel:**

The phased haplotype file from the 1000 Genomes phase 3 reference panel (1KGP3-ALL) data for chromosome 20 was converted from HAP/LEGEND/SAMPLE format to HAPS/SAMPLE format by editing the first 5 columns. The phased haplotypes for South Asian Ancestry (SAS) subpopulations were extracted and then converted into reference panel by the commands as given below:

**Extract phased haplotypes from SAS samples from full panel:**

shapeit -convert \

--input-haps 1000GP_Phase3_chr20_haps 1000GP_Phase3.sampleID.txt \

--exclude-ind 1000GP_Phase3.sample_nonSASsamples.txt \

--output-log 1000GP_Phase3_chr20_haps.phasedSAS.log \

--output-haps 1000GP_Phase3_chr20_haps.phasedSAS \

--thread 8

**Convert phased haplotypes into reference panel:**

./shapeit -convert \

--input-haps 1000GP_Phase3_chr20_haps.phasedSAS \

--output-log 1000GP_Phase3_SAS_chr20_Ref.log \

--output-ref 1000GP_Phase3_SAS_chr20_Ref.haps 1000GP_Phase3_SAS_chr20_Ref.legend.gz 1000GP_Phase3_SAS_chr20_Ref.sample \

--thread 4

**Supplementary Note 2:**

**Pre-phasing and imputation commands:**

**Pre-phase genome-wide array data:**

shapeit -B affy1880_hg19.chr20 \

-M genetic_map_chr20_combined_b37.txt \

-O affy1880_hg19.chr20.phased \

--thread 8

--output-log affy1880_hg19.chr20.log

**Convert phased haplotypes into reference panel:**

shapeit -convert \

--input-haps WIP407_hg19.chr20.phased \

--output-log WIP407_hg19.chr20_Ref.log \

--output-ref WIP407_hg19.chr20_Ref.haps WIP407_hg19.chr20_Ref.legend WIP407_hg19.chr20_Ref.sample

**Merge 1KGP3-ALL and Western Indian Reference (WIP) panels:**

impute2 \

-allow_large_regions

-merge_ref_panels

-include_buffer_in_output \

-m genetic_map_chr20_combined_b37.txt \

-h 1000GP_Phase3_chr20.hap.gz WIP407_hg19.chr20_Ref.haps.gz \

-l 1000GP_Phase3_chr20.legend.gz WIP407_hg19.chr20_Ref.legend.gz \

-Ne 20000 \

-k_hap 3000 \

-buffer 1000kb \

-int 1 63025520 \

-merge_ref_panels_output_ref 1KGP3ALL_WIP407_hg19.chr20_Ref

**Imputation:**

impute2

-filt_rules_l 'SAS<0.0005' 'TYPE!=Biallelic_SNP' \

-use_prephased_g -known_haps_g affy1880_hg19.chr20.phased.haps \

-sample_g affy1880_hg19.chr20.phased.sample \

-m genetic_map_chr20_combined_b37.txt \

-h 1000GP_Phase3_chr20.hap.gz \

-l 1000GP_Phase3_chr20.legend.gz \

-exclude_samples_g samplesIDs_toExcludefromImputation.txt \

-Ne 20000 \

-k_hap 3000 \

-buffer 1000kb \

-int 1 5000000 \

-o affy1880_Ph3_chr20_3khp_001.impute2
